# Dietary Lutein Plus Zeaxanthin Intake and *DICER1* rs3742330 A > G Polymorphism Relative to Colorectal Cancer Risk

**DOI:** 10.1038/s41598-019-39747-5

**Published:** 2019-03-04

**Authors:** Jimi Kim, Jeonghee Lee, Jae Hwan Oh, Hee Jin Chang, Dae Kyung Sohn, Oran Kwon, Aesun Shin, Jeongseon Kim

**Affiliations:** 1 Department of Cancer Biomedical Science, Graduate School of Cancer Science and Policy, National Cancer Center, Goyang, South Korea; 2 Center for Colorectal Cancer, National Cancer Center Hospital, National Cancer Center, Goyang, South Korea; 3 Department of Nutritional Science and Food Management, Ewha Womans University, Seoul, South Korea; 4 Department of Preventive Medicine, Seoul National University College of Medicine, Seoul, South Korea

## Abstract

It is unclear whether dietary lutein/zeaxanthin intake in colorectal cancer is associated with microRNA processing involved in *DICER1* cleavage for messenger RNA translation. We investigated whether dietary lutein/zeaxanthin intake affects colorectal cancer risk in patients with a *DICER1* rs3742330 polymorphism. In this hospital-based case-control study, we recruited 923 colorectal cancer patients and 1,846 controls based on eligibility criteria, a semiquantitative food frequency questionnaire and the *DICER1* rs3742330 genotype. Odds ratios (ORs) and 95% confidence intervals (CIs) were calculated by unconditional logistic regression adjusted for confounders. The highest quartile of lutein/zeaxanthin consumption was inversely associated with a reduced colorectal cancer risk (OR, 95% CI = 0.25, 0.18–0.36). Carrying G allele (AG + GG) showed a significantly reduced colorectal cancer incidence compared with that of AA carriers (OR, 95% CI = 0.71, 0.55–0.91). Those carrying the G allele (AG + GG) along with high lutein/zeaxanthin consumption were markedly associated with a decreased colorectal cancer risk (OR, 95% CI = 0.32, 0.22–0.46, *P* for interaction = 0.018), particularly for rectal cancer (OR, 95% CI = 0.24, 0.15–0.39, *P* for interaction = 0.004), compared with that of AA carriers with low lutein/zeaxanthin intakes. In conclusion, colorectal cancer risk was related to an interactive effect between dietary lutein/zeaxanthin intake and the *DICER1* rs3742330 polymorphism.

## Introduction

Colorectal cancer (CRC) is a common cancer that causes mortality worldwide, with approximately 1.4 million new cases and nearly 694,000 deaths in 2012^1^. Cancer incidence and mortality data for 2018 indicated that CRC is one of the most common cancers in Korea and has an age-standardized incidence rate of 23.1 per 100,000 people in both sexes worldwide^2^. Risk factors for CRC include age (>50), history of adenomatous polyps, family history of CRC, inherited genetic risk, physical activity, obesity, smoking, alcohol consumption, and dietary habits^3^. A leading cause of CRC is a western-style diet, which includes low fruit and vegetable intake and is an important risk factor in developing colorectal carcinogenesis^3,4^.

Lutein/zeaxanthin are abundant xanthophyll carotenoids in many dark-green leafy vegetables and in egg yolks^5^. Lutein/zeaxanthin protect against degenerative eye conditions including age-related macular degeneration and cataracts^6^. Several studies have explored the association between lutein/zeaxanthin and cancer, showing that they provide potential antioxidant functions in cellular mechanisms that regulate apoptosis^7–9^, modulate gap junctional intercellular communication^10^, and stimulate DNA strand break repair^11^. Epidemiology studies have shown that lutein/zeaxanthin intake is inversely associated with breast, lung and CRC development^12^. However, no evidence exists to support an interaction between dietary lutein/zeaxanthin intake and genetic variants associated with a risk of developing CRC.

Many investigators have revealed the importance of microRNAs (miRNAs) in cancer initiation and progression via their effects in silencing tumor suppressive and oncogenic messenger RNAs (mRNAs)^13^. miRNAs are small noncoding regions that are linked to both mRNA translation and RNA silencing^14^. The *DICER1* gene is an endonuclease with RNase III activity that cleaves pre-miRNA as part of a trans activating response RNA binding protein (TRBP) complex to generate miRNA and small interfering RNA^15^. *DICER1* is involved in the processing necessary for miRNA biogenesis. In cytoplasmic processing, Dicer is an essential enzyme for miRNA biogenesis and maturation, enabling mRNA to repress gene expression^16^. The miRNAs processed by *DICER1* have been reported to mediate ovarian cancer, breast cancer, and CRC through carcinogenic mechanisms such as cellular proliferation, apoptosis, and differentiation^17,18^. A single nucleotide polymorphism (SNP) of rs3742330, which is related to miRNA synthesis, is located in the 3′-untranslated region (UTR) of *DICER1* on 14q32, and its regulation has become important in association with CRC risk^19,20^. However, limited evidence indicates that dietary factors may be associated with regulating *DICER1* rs3742330 in CRC cases.

In this study, we analyzed *DICER1* rs3742330 genotypes from DNA samples and evaluated the intake of lutein/zeaxanthin by using a validated semiquantitative food frequency questionnaire (SQFFQ) among controls and CRC patients to identify CRC etiology. This study investigated whether lutein/zeaxanthin intake shows an interaction with *DICER1* rs3742330 in relation to CRC risk.

## Results

### General characteristics of the study participants

Table 1 shows the study subjects’ demographic characteristics. With respect to sociodemographic factors, cases were statistically associated with education level, professional occupation, income and positive first-degree family history of CRC (*P* < 0.001). Patients had a higher rate of ex-alcohol drinkers and physical inactivity than the controls (*P* < 0.001). No significant differences were observed for BMI or smoking status. The mean total energy intakes for the controls and the CRC patients were 1,689.6 ± 560.43 kcal/day and 2,2026.3 ± 533.96 kcal/day, respectively (*P* < 0.001). The mean lutein/zeaxanthin intake was 3.61 ± 2.91 mg/day for the controls and 2.72 ± 1.78 mg/day for the CRC patients (*P* < 0.001).

**Table 1:** General characteristics of subjects.

	Controls (n = 1,846)	Cases (n = 923)	*P*-value^a^
Age (years)			
Mean ± SD	56.09 ± 9.12	56.58 ± 9.71	0.20
Sex (n, %)			
Male	1250 (67.7)	625 (67.7)	>0.99
Female	596 (32.3)	298 (32.3)	
Body mass index (BMI, kg/m^2^) (n, %)			
<25	1226 (66.4)	640 (69.3)	0.12
≥25	620 (33.6)	283 (30.7)	
Education level (n, %)			
Middle school or less	282 (15.6)	321 (34.8)	<0.001
High school	587 (32.6)	369 (40.0)	
College or more	934 (51.8)	233 (25.2)	
Occupation (n, %)			
Professionals, Administrative, Management, Office jobs	481 (26.4)	189 (20.5)	<0.001
Sales and Service positions	403 (22.1)	38 (4.1)	
Agriculture, Manufacturing, Mining, Army service	241 (13.2)	141 (15.3)	
Housekeeping, Unemployment, and Others	698 (38.3)	555 (60.1)	
Income (10,000 won/month) (n, %)			
<200	388 (23.0)	321 (34.8)	<0.001
200-400	754 (44.7)	387 (41.9)	
>400	545 (32.3)	215 (23.3)	
Smoking status (n, %)			
None	818 (44.3)	409 (44.3)	0.16
Ex-smoker	687 (37.2)	318 (34.5)	
Current-smoker	341 (18.5)	196 (21.2)	
Alcohol consumption (n, %)			
None	560 (30.3)	279 (30.2)	<0.001
Ex-drinker	169 (9.2)	129 (14.0)	
Current-drinker	1117 (60.5)	515 (55.8)	
Physical activity (n, %)			
Yes	1047 (58.2)	311 (33.7)	<0.001
No	753 (41.8)	612 (66.3)	
First-degree family history of CRC (n, %)			
Yes	99 (5.4)	86 (9.3)	<0.001
No	1743 (94.6)	837 (90.7)	
Total caloric intake (kcal/day)			
Mean ± SD	1689.60 ± 560.43	2026.34 ± 533.96	<0.001
Lutein/zeaxanthin intake (mg/day)			
Mean ± SD^b^	3.61 ± 2.91	2.72 ± 1.78	<0.001
Median (IQR)^c^	2.86 (1.95, 4.35)	2.32 (1.74, 3.11)	

^a^*P*-values were calculated using the χ^2^ test for categorical variables and the *t*-test for continuous variables. ^b^Lutein/zeaxanthin intake was adjusted for total energy intake using the residual method. ^c^IQR, interquartile ranges.

### Association of dietary lutein/zeaxanthin intake with CRC risk

Table 2 shows the ORs and 95% CIs of CRC risk for lutein/zeaxanthin intake. Dietary lutein/zeaxanthin intake on CRC risk was compared by quartile groups. Lutein/zeaxanthin intake was strongly significantly associated with CRC risk in both crude and multivariable models adjusted for age, sex, BMI, education level, occupation, income, alcohol consumption, physical activity, first-degree family history of CRC, and total energy intake. The highest lutein/zeaxanthin intake quartile was inversely associated with CRC risk compared with that of the lowest quartile group (OR, 95% CI = 0.25, 0.18–0.36, highest versus lowest quartile, *P* for trend < 0.001). When stratified by anatomical subsite for CRC, the highest lutein/zeaxanthin intake showed significant inverse associations in proximal, distal, and rectal cancer compared with those of lower lutein/zeaxanthin intake (proximal colon cancer OR, 95% CI = 0.38, 0.21–0.71; distal colon cancer OR, 95% CI = 0.21, 0.12–0.35; rectal cancer OR, 95% CI = 0.26, 0.17–0.41, highest versus lowest quartile, *P* for trend < 0.001).

**Table 2:** Association of lutein/zeaxanthin intake with CRC risk stratified by anatomic subsite.

Lutein/zeaxanthin (mg/day)	Q1 (<1.95)	Q2 (1.95-<2.86)	Q3 (2.86-<4.35)	Q4 (≥4.35)	*P* for trend
CRC					
No. Controls/Cases	462/305	461/325	461/205	462/88	
Crude OR (95% CI)	1.0 (ref)	1.07 (0.87, 1.31)	0.67 (0.54, 0.84)	0.29 (0.22, 0.38)	<0.001
Multivariable OR (95% CI)^a^	1.0 (ref)	1.00 (0.76, 1.31)	0.66 (0.49, 0.88)	0.25 (0.18, 0.36)	<0.001
Proximal colon cancer					
No. Controls/Cases	462/49	461/56	461/40	462/20	
Crude OR (95% CI)	1.0 (ref)	1.15 (0.76, 1.72)	0.82 (0.53, 1.27)	0.41 (0.24, 0.70)	<0.001
Multivariable OR (95% CI)^a^	1.0 (ref)	1.11 (0.69, 1.78)	0.85 (0.51, 1.42)	0.38 (0.21, 0.71)	<0.001
Distal colon cancer					
No. Controls/Cases	462/95	461/104	461/69	462/26	
Crude OR (95% CI)	1.0 (ref)	1.10 (0.81, 1.49)	0.73 (0.52, 1.02)	0.27 (0.17, 0.43)	<0.001
Multivariable OR (95% CI)^a^	1.0 (ref)	0.91 (0.63, 1.33)	0.66 (0.44, 0.99)	0.21 (0.12, 0.35)	<0.001
Rectal cancer					
No. Controls/Cases	462/151	461/158	461/93	462/42	
Crude OR (95% CI)	1.0 (ref)	1.05 (0.81, 1.36)	0.62 (0.46, 0.82)	0.28 (0.19, 0.40)	<0.001
Multivariable OR (95% CI)^a^	1.0 (ref)	1.00 (0.72, 1.39)	0.59 (0.41, 0.86)	0.26 (0.17, 0.41)	<0.001

^a^Multivariable odds ratio (OR) was adjusted for age, sex, BMI, education level, occupation, income, alcohol consumption, physical activity, first-degree family history of CRC, and total energy intake. Q, quartile.

### Association of *DICER1* rs3742330 variant with CRC risk

Table 3 shows the ORs and 95% CIs of CRC risk according to the *DICER1* rs3742330 variant. The *DICER1* rs3742330 minor allele frequency was 0.44. Polymorphism in the controls was in Hardy-Weinberg equilibrium (HWE). Heterozygous AG among the cases was significantly associated with CRC risk in multivariable models adjusted for the covariates mentioned above (OR, 95% CI = 0.70, 0.53–0.91, *P* < 0.01). When comparing the rs3742330 allele frequencies, carrying a G allele (AG + GG) significantly decreased the risk of CRC compared with those who were AA homozygous via a dominant model (OR, 95% CI = 0.71, 0.55–0.91, *P* < 0.01).

**Table 3:** Association between *DICER1* (rs3742330) polymorphism and CRC risk.

*DICER1* (rs3742330)	No. Controls (%)	No. Cases (%)	Crude OR (95% CI)	*P*-value^a^	Multivariable OR (95% CI)^b^	*P*-value^a^
AA	435 (31.1)	244 (34.9)	1.0 (ref)		1.0 (ref)	
AG	697 (49.8)	325 (46.4)	0.83 (0.68, 1.02)	0.08	0.70 (0.53, 0.91)	<0.01
GG	268 (19.1)	131 (18.7)	0.87 (0.67, 1.13)	0.30	0.73 (0.52, 1.03)	0.08
Dominant						
AA	435 (31.1)	244 (34.9)	1.0 (ref)		1.0 (ref)	
AG + GG	965 (68.9)	456 (65.1)	0.84 (0.70, 1.02)	0.08	0.71 (0.55, 0.91)	<0.01
Recessive						
AA + AG	1132 (80.9)	569 (81.3)	1.0 (ref)		1.0 (ref)	
GG	268 (19.1)	131 (18.7)	0.97 (0.77, 1.23)	0.81	0.91 (0.68, 1.23)	0.54

Successful rs3742330 genotyping was performed with A > G, 1,400 controls and 700 cases. ^a^*P*-values were calculated using the χ^2^ test. ^b^Multivariable odds ratio (OR) was adjusted for age, sex, BMI, education level, occupation, income, alcohol consumption, physical activity, first-degree family history of CRC, and total energy intake.

### Association of dietary lutein/zeaxanthin intake with CRC risk, stratified by *DICER1* rs3742339 variant

Table 4 shows an interaction between lutein/zeaxanthin intake and the *DICER1* rs3742330 variant with CRC risk. Lutein/zeaxanthin intake was categorized into low and high groups, and CRC was stratified by anatomic subsite. High lutein/zeaxanthin intake while carrying a G allele (AG + GG) showed a significant interaction with low CRC risk compared with that of AA homozygous patients (OR, 95% CI = 0.32, 0.22–0.46, *P* for interaction = 0.018, AG + GG carriers with high intake vs. AA carriers with low intake). When CRC was stratified by anatomic subsite, lutein/zeaxanthin intake was inversely associated with rectal cancer (OR, 95% CI = 0.24, 0.15–0.39, *P* for interaction = 0.004, AG + GG carrier with high intake vs. AA carriers with low intake).

**Table 4:** Interaction between lutein/zeaxanthin intake and *DICER1* (rs3742330) polymorphism in relation to CRC risk stratified by anatomic subsite.

*DICER1* (rs3742330)	CRC		Proximal colon cancer		Distal colon cancer		Rectal cancer	
	Low	High	Low	High	Low	High	Low	High
No. Controls/Cases								
AA	219/156	216/88	219/24	216/13	219/50	216/28	219/79	216/46
AG + GG	481/322	484/134	481/61	484/27	481/98	484/53	481/155	484/53
Crude OR (95% CI)								
AA	1.0 (ref)	0.57 (0.42, 0.79)	1.0 (ref)	0.55 (0.27, 1.11)	1.0 (ref)	0.57 (0.35, 0.94)	1.0 (ref)	0.59 (0.39, 0.89)
AG + GG	0.94 (0.73, 1.21)	0.39 (0.29, 0.52)	1.16 (0.70, 1.91)	0.51 (0.29, 0.90)	0.89 (0.61, 1.30)	0.48 (0.32, 0.73)	0.89 (0.65, 1.22)	0.30 (0.21, 0.45)
*P* for interaction	0.11		0.61		0.86		0.041	
Multivariable OR (95% CI)^a^								
AA	1.0 (ref)	0.67 (0.44, 1.02)	1.0 (ref)	0.56 (0.25, 1.24)	1.0 (ref)	0.57 (0.31, 1.03)	1.0 (ref)	0.76 (0.45, 1.27)
AG + GG	0.91 (0.66, 1.25)	0.32 (0.22, 0.46)	1.09 (0.61, 1.95)	0.46 (0.24, 0.89)	0.90 (0.57, 1.41)	0.42 (0.25, 0.68)	0.84 (0.56, 1.24)	0.24 (0.15, 0.39)
*P* for interaction	0.018		0.59		0.59		0.004	

Lutein/zeaxanthin intake was categorized into low and high groups based on the median level of their control group’s intake (lutein/zeaxanthin = 2.81 mg/day). ^a^Multivariable odds ratio (OR) was adjusted for age, sex, BMI, education level, occupation, income, alcohol consumption, physical activity, first-degree family history of CRC, and total energy intake.

## Discussion

The present study showed an association between dietary lutein/zeaxanthin intake and CRC risk among those with the *DICER1* rs3742330 genotype in a Korean population. A high intake of lutein/zeaxanthin was inversely associated with CRC risk in G allele carriers (AG + GG) compared with that in AA homozygous *DICER1* rs3742330 carriers.

Previous epidemiological studies have shown conflicting data regarding the association between lutein/zeaxanthin intake and CRC risk. Case-control studies have shown that CRC and lutein/zeaxanthin intake, either individually^21,22^ or together^23^, are significantly inversely associated. In a pooled analysis of cohort studies, dietary lutein/zeaxanthin intake was associated with a slightly reduced CRC risk^24^, and a similar association was found for the risk of colorectal adenomas in a cohort of male health professionals^25^. Because dietary intake affects lutein/zeaxanthin serum levels, these levels, either alone or together, were decreased in patients with gastrointestinal cancer^26^, colorectal polyps^27,28^, and colorectal neoplasms^29^ compared with those in controls. In contrast, several studies reported that lutein/zeaxanthin intake was not associated with CRC risk. Six case-control studies showed that dietary intake or serum levels of either lutein only^30–32^ or lutein/zeaxanthin^33–35^ were not associated with CRC risk. Several large prospective cohort studies have supported the lack of an association between dietary lutein/zeaxanthin intake and CRC^36,37^. These results might be attributed to differences in the range of lutein/zeaxanthin intake, CRC status and the point that CRC examination began in the cohort study. In an *in vitro* study, dietary lutein inhibited colonic aberrant crypt focal development in rats, suggesting that it may help prevent colon carcinogenesis^38,39^. Other studies have suggested that lutein/zeaxanthin bioavailability affects carcinogenesis via its antioxidant properties by inducing apoptosis and proliferation, preventing oxygen radicals, regulating gene expression, and activating the immune response^6,12,21,40^. In this study, high lutein/zeaxanthin intake was strongly inversely associated with reduced CRC risk in all anatomic subsites, including the proximal and distal colon and rectum.

*DICER1* plays a role in a biogenesis pathway that is known to regulate the repression of mRNA translation and trigger mRNA degradation by binding to the 3′-untranslated region (UTR) of target mRNA. Loss of function in heterozygous *DICER1* germline pathogenic variants and somatic missense variants of *DICER1* result in the abnormal production of miRNAs^41–45^. Abnormal miRNA expression is suspected to promote cellular carcinoma by affecting proliferation, apoptosis, mitosis, and cell-cycle progression^46,47^. SNPs in the 3′-UTR have been reported to contribute to the regulation of transcript stabilization^48^. The 3′-UTR is considered a place of pathological and polymorphic sites related to disease involving miRNA activation^49^. A growing body of evidence has indicated that the up- and downregulation of *DICER1* are related to the development of tumorigeneses such as lung, breast, ovarian, skin, prostate cancers, and CRC via the alteration of miRNA expression^17,20,50^. Moreover, with respect to *DICER1* related cancers, several studies have suggested that loss of function or mutation of *DICER1* may affect stem cell proliferation, differentiation, and cell fate and induce embryonal or blastoma carcinomas^17,51,52^. In an *in vivo* study, inactivating *Dicer1* in a mouse model showed that it functioned as a haploinsufficient tumor suppressor in retinoblastoma and promoted hepatocarcinogenesis^53,54^.

Several studies have explored the deregulation of *DICER1*, which is required for miRNA processing, as part of CRC etiology. Dicer mRNA levels were significantly increased in CRC, particularly in rectal cancer, compared with those in normal mucosa^55^. The downregulation of Dicer was found to be a prognostic indicator underlying tumor development in CRC^56^. The overexpression of Dicer was associated with shorter survival time in CRC patients^57^. In an *in vivo* study, impaired DICER1 function indicated a stemness phenotype and metastasis in colon cancer^58^. Taken together, evidence has shown that the interaction between *DICER1* and CRC demonstrates the importance of miRNA-related SNPs (miR-SNPs), which alter miRNA levels and thereby influence mRNA transcription, in *DICER1*.

In recent years, functional studies have shown that miRNAs can be oncogenes or tumor suppressor genes depending on their target genes^59,60^. The SNP rs3742330 located in the 3′-UTR of *DICER1* has been reported to be the target site of two miRNAs, miR-3622a-5p and miR-5582-5p^61,62^. The miR-SNP rs3742330 in *DICER1* contributes to T-cell lymphoma, renal cell carcinoma, oral premalignant lesions, and CRC^19,63–65^. The variant allele of *DICER1* rs3742330 is associated with CRC despite inconsistent patterns in recent literature. Zhao *et al*. reported that the AA allele of *DICER1* rs3742330 is related to an increased CRC risk^19^, while Cho *et al*. determined that the *DICER1* rs3742330 AG genotype leads to an increased risk of colon cancer but not rectal cancer^66^. Our findings indicated that *DICER1* rs3742330 AG heterozygotes were associated with an increased risk of CRC. Moreover, when comparing the frequency of the *DICER1* rs3742330 allele, carrying a G allele (AG + GG) was significantly associated with a decreased risk of CRC compared with AA homozygous carriers via a dominant model. However, when stratified by anatomical sites, *DICER1* rs3742330 was significantly associated with rectal cancer. These reports and our findings suggest that *DICER1* rs3742330 may impact the regulation of *DICER1* expression, even if it shows a tumor-specific pattern, which requires laboratory-based functional studies.

In this study, high lutein/zeaxanthin intake while carrying a G allele (AG + GG) for *DICER1* rs3742330 showed a significant interaction, thus resulting in a reduced CRC risk compared with that for low lutein/zeaxanthin intake among AA homozygous individuals. Our study suggests several potential mechanisms that account for the interaction between lutein/zeaxanthin and *DICER1* rs3742330 regarding CRC risk. First, the function of *DICER1*, influenced by rs3742330 plays a pivotal role in CRC tumorigenesis by altering the expression of miRNA-related oncogenic pathways underlying cellular transformation, such as proliferation, apoptosis, invasion, and metastasis^67^. Regarding miRNA expression and CRC risk, Slattery *et al*. suggested that dietary and lifestyle factors related to inflammation and oxidative stress are linked to regulating miRNA expression in colorectal tissue, leading to an elevated CRC risk^68^. Although we did not measure miRNA expression levels by lutein/zeaxanthin intake, our study supports the notion that dietary lutein/zeaxanthin intake impacts *DICER1* activity by regulating miRNA expression, thus affecting CRC risk via an underlying interaction between antioxidant effects and miRNA expression. Second, DNA damage is a possible cause of tumorigenesis^69^. Several studies have identified that a loss of Dicer contributes to tumorigenesis progression via DNA damage^69,70^. Among various nutrients related to DNA damage, Haegele *et al*. proposed an inverse association between plasma lutein and oxidative indices, showing that lutein affects DNA damage repair^71^. Lutein may influence the DNA damage repair associated with Dicer’s role in CRC risk. Third, deleting *Dicer* reduces T-reg cell numbers and immune pathology, indicating Dicer’s importance in immune regulation and immune cell development^72,73^. Several studies have reported that lutein/zeaxanthin action induces cell-mediated and humoral immune responses^12,40^. Because lutein/zeaxanthin exert immunological properties via the immune system, an interaction between lutein/zeaxanthin and Dicer could reduce CRC risk via an underlying immune system effect. Despite numerous studies investigating the role of the miRNA associated *DICER1* in CRC risk, the interaction between *DICER1* functions and other elements, such as diet and environmental factors resulting in CRC, remain undetermined.

To our knowledge, this is the first report to examine the miRNA associated *DICER1* gene and dietary interaction as part of CRC etiology. Dietary lutein/zeaxanthin intake and the *DICER1* rs3742330 genotype were inversely associated in CRC, providing new insight into a protective effect of lutein/zeaxanthin against CRC risk in patients carrying the G allele. However, this study has limitations that must be considered when interpreting our findings. This study was designed as a hospital-based case-control study and may have been affected by selection bias. To evaluate dietary lutein/zeaxanthin intake, we used a validated SQFFQ that may have been affected by recall bias among the participants. In addition, we analyzed the *DICER1* rs3742330 genotype to determine the association between lutein/zeaxanthin intake and CRC risk. Further investigation regarding how lutein/zeaxanthin impacts miRNA associated *DICER1* expression levels and miRNA processing machinery relative to CRC risk is needed to understand the associated molecular mechanisms.

The results of this study indicate that lutein/zeaxanthin affects miRNA processing by regulating *DICER1*, which appears to result in a lower CRC risk. Furthermore, rs3742330 may either directly or indirectly alter Dicer-mediated miRNA levels, which can influence mRNA transcription and CRC risk. We propose that future studies determine how dietary lutein/zeaxanthin intake is associated with regulating *DICER1* rs3742330 and the underlying molecular mechanism in CRC risk.

## Methods

### Study population

This was a hospital-based case-controlled study in a Korean population designed to detect associations between dietary intake, genetic factors, and CRC risk, which is described elsewhere^4^. In brief, this study by the Center for Colorectal Cancer of the National Cancer Center Korea included 1,070 patients newly diagnosed with colorectal tumors pathologically confirmed as adenocarcinoma by endoscopic biopsies who agreed to participate between August 2010 and August 2013. The 14,201 cancer-free controls, confirmed by linking to the Korea Central Cancer Registry (KCCR) database, were recruited by the Center for Cancer Prevention and Detection at the National Cancer Center Korea for a health check-up program provided by the National Health Insurance Cooperation between October 2007 and December 2017. Based on eligibility criteria, we excluded 5,189 subjects (145 cases and 5,044 controls) with an incomplete SQFFQ and 122 subjects (2 cases and 120 controls) with an implausible self-reported energy intake (<500 or >4,000 kcal/day). Of the remaining subjects, controls were matched to cases at a 1:2 ratio (cases: controls) by sex within a 5-year age range. For genetic analysis, patients with missing blood samples were excluded (Supplementary Fig. S1). This study conformed to the National Cancer Center Korea guidelines, and participants signed a written informed consent document. The Institutional Review Board of the National Cancer Center Korea approved this study (IRB Nos. NCCNCS-10-350 and NCC2015-0202).

### Data collection

Demographic characteristics and lifestyle information were obtained from a structured questionnaire administered by a trained interviewer. Participants were also asked about their dietary intake using a 106-item SQFFQ that was previously determined to be reliable and valid^74^. Energy intake and individual nutrients were calculated using Computer Aided Nutritional Analysis Program 4.0 (CAN-PRO 4.0, The Korean Nutrition Society, Seoul, Korea). To estimate lutein/zeaxanthin intake, we used a carotenoid database developed by merging databases from the United States Department of Agriculture, the Korea functional food composition table, and literature searches^75,76^. From the 2,903-items in the carotenoid database, we estimated lutein/zeaxanthin intake to identify its association with CRC risk.

### SNP genotyping

Genomic DNA was extracted from participants’ blood samples following the manufacturer’s instructions, using the MagAttract DNA Blood M48 Kit (Qiagen, Hilden, Germany) and BioRobot M48 automatic extraction equipment (Qiagen). SNP genotyping was performed using the MassARRAY iPLEX Gold Assay (Agena Bioscience, San Diego, CA, USA). The rs3742330 genotyping results were A > G in 1,400 controls and 700 cases.

### Statistical analysis

Demographic and lifestyle characteristics were analyzed by χ^2^ tests for categorical variables and Student’s *t*-test for continuous variables. Dietary lutein/zeaxanthin intake was adjusted for total energy intake using the residual method^77^. To compare its association with CRC risk, dietary lutein/zeaxanthin intake was evaluated by exposure quartiles in the control intake. ORs and 95% CIs were calculated using unconditional logistic regression models, adjusting for age, sex, BMI, education level, occupation, income, alcohol consumption, physical activity, first-degree family history of CRC, and total energy intake. We analyzed CRC risk stratified by anatomical subsite (proximal colon, distal colon, and rectal cancer) using a multinomial logistic regression model. The rs3742330 was in Hardy-Weinberg equilibrium (HWE), and the genotype was categorized into dominant (AA vs. AG + GG) and recessive (AA + AG vs. GG) effect models. To examine diet-gene interaction, ORs and 95% CIs were estimated based on the rs3742330 genetic model and lutein/zeaxanthin intake categories using a multiple logistic regression model, adjusting for the aforementioned covariates. We divided the participants into two intake groups (low/high) based on median levels of control to test the interaction between lutein/zeaxanthin intake and the rs3742330 genetic model using a likelihood ratio. All analyses were estimated using SAS version 9.4 software (SAS Institute Inc., Cary, NC, USA). A significance level of less than 0.05 was used and determined with two-tailed tests.

## Supplementary information

Supplementary Figure S1. Flow chart of subject recruitment.
