# Supplementary material for: Dietary Lutein Plus Zeaxanthin Intake and DICER1 rs3742330 A > G Polymorphism Relative to Colorectal Cancer Risk
Source: Sci Rep. 2019 Mar 4;9:3406. doi: 10.1038/s41598-019-39747-5 (PMC6399314; doi:10.1038/s41598-019-39747-5)

Supplementary materials

Dietary Lutein Plus Zeaxanthin Intake and *DICER1* rs3742330 A > G Polymorphism Relative to Colorectal Cancer Risk

Author names and affiliations:

Jimi Kim[1], Jeonghee Lee[1], Jae Hwan Oh[2], Hee Jin Chang[2], Dae Kyung Sohn[2], Oran Kwon[3], Aesun Shin[4], and Jeongseon Kim[1]*

[1]Department of Cancer Biomedical Science, Graduate School of Cancer Science and Policy, National Cancer Center, Goyang, South Korea;

[2]Center for Colorectal Cancer, National Cancer Center Hospital, National Cancer Center, Goyang, South Korea;

[3]Department of Nutritional Science and Food Management, Ewha Womans University, Seoul, South Korea;

[4]Department of Preventive Medicine, Seoul National University College of Medicine, Seoul, South Korea

***Correspondence to:**

Jeongseon Kim, PhD, Department of Cancer Biomedical Science, Graduate School of Cancer Science and Policy, National Cancer Center, Goyang-si, 10408, Gyeonggi-do, South Korea. Tel: +82-31-920-2570; Fax: 82-31-920-2579; E-mail: jskim@ncc.re.kr

Supplementary Figure S1. Flow chart of subject recruitment.

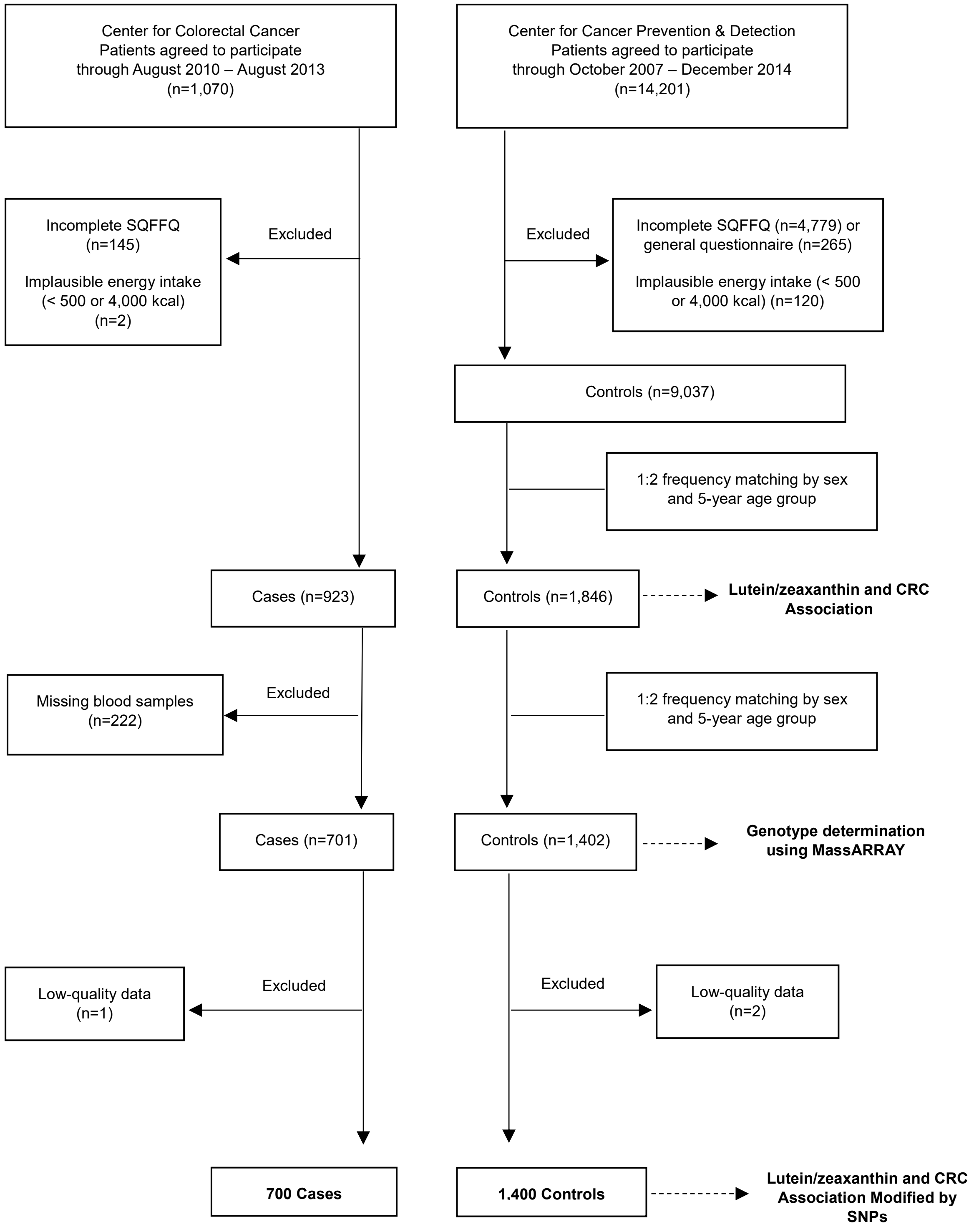

Supplement: Supplementary file 1 — Supplementary Figure S1. Flow chart of subject recruitment. [file 41598_2019_39747_MOESM1_ESM.pdf]
